# Anastomosis Groups of *Rhizoctonia solani* associated with tomato foot rot in Pothohar Region of Pakistan

**DOI:** 10.1038/s41598-019-40043-5

**Published:** 2019-03-07

**Authors:** Amjad Shahzad Gondal, Abdul Rauf, Farah Naz

**Affiliations:** Department of Plant Pathology, PMAS Arid Agriculture University Rawalpindi, Rawalpindi, Pakistan

## Abstract

*Rhizoctonia solani* Kühn (teleomorph = *Thanatephorus cucumeris* (Frank) Donk) is one of the important soil-borne fungal pathogen, which infects tomato with typical symptoms of seedling damping-off and foot rot. During surveys (2014 and 2015 crop season) of nine tomato growing areas in Pothohar region of Pakistan, symptoms of foot rot were noted on approximately 33.4% of the plants observed at soil line level of the stem. Lesions on infected plant stems were irregular in shape, water-soaked, brown in colour manifesting sunken appearance. Fungal colonies isolated from stem portions of the diseased plants on malt extract agar medium were light grey to brown in colour with abundant mycelial growth and branched hyphae. A septum was always present in the branch of hyphae near the originating point with a slight constriction at the branch. No conidia or conidiophores were observed. All isolates were multinucleate when subjected to DAPI (4′,6-diamidino-2-phenylindole) stain. Based on morphological characteristics of fungal hyphae, isolates were identified as *R. solani*. Restriction analysis of PCR-amplified ribosomal DNA with four discriminant enzymes (*MseI, AvaII, HincII*, and *MunI*) and hyphal interactions with known tester strains confirmed these isolates belong to AG-3-PT (64.2%), AG-2-1 (14.2%), AG-2-2 (9.5%), AG-5 (7.1%) and AG-4-HGI (4.7%). AG-3-PT was widely distributed to major tomato growing areas while other groups were confined to distinct locations. Internal transcribed spacer (ITS) region of rDNA was amplified with the primers ITS1/ITS4 and sequenced which had 99–100% identity with the corresponding gene sequences of respective *R. solani* AGs. To confirm Koch’s postulates, four week old tomato plants were transplanted into 1.5 L plastic pots containing sterilized potting mixture i.e. sand: clay: farmyard manure, at the rate of 1:1:1. Soil inoculum containing 10 g of barley grains colonized with each isolate of *R. solani* for 14 days was mixed in the upper 2 cm layer of soil (Taheri and Tarighi, 2012). A set of uninoculated plants was used as a control. Ambient conditions were provided under the greenhouse. 21 days after inoculation, water-soaked greyish to brown lesions similar to the symptoms of the previous infection were observed on stem portions of all inoculated plants while control plants remained symptomless. Fungus re-isolated from infections was confirmed as *R. solani* by microscopic appearance of the hyphae. Present study is the first report of AG composition of *R. solani* infecting tomato in Pakistan which will be useful to breeding programs working on varietal evaluation.

## Introduction

*Solanum lycopersicum* L., formerly known as *Lycopersicon esculentum* Mill., is one of the dominant vegetable crop worldwide that is generally cultivated in warm or tropical climate. It is an adaptable crop used for both fresh market and processing in prepared foods as canned, ketchup, sauce, juice, paste, powder, puree, salad dressings, soups, vegetable and juice cocktails, frozen tomatoes, preserved or dried foods. It is now considered to be a part of the daily diet^1^. Tomato is the second most consumed vegetable after potato^2^. In Pakistan tomato is grown on an area of 62536 hectares with a production of about 587111 tons and yield 10.5 tons/ha (FAO, 2017). The Pothohar region contributes 7.58% of the total tomato production of the country. This local tomato yield is low as compared to other countries of the world including USA (97.9 tons/ha), China (58 tons/ha) and India (23.4 tons/ha)^3^.

Several biotic and abiotic factors contribute to this low tomato yield. Of all primary food crops, tomato crop also endures the utmost yield losses and a significant part of the produce is lost due to disease attack and pests^4–6^. Among the biological factors causing disease in tomato crop, fungal pathogens especially *R. solani* is the worst damaging that play a key role in reducing the yield^7–9^.

*R. solani* has a wide host range of more than 200 plant species especially Solanaceae family including eggplant, pepper, potato, tobacco and tomato cultivated under both, greenhouse and field conditions^10–12^. *R. solani* is a species complex of several anastomosis groups (AGs) based on the hyphal fusion of isolates which differ in genotypic and phenotypic characters^13^. To date, thirteen AGs designated as AG1–AG13 and AG-BI have been assigned on the basis of hyphal anastomosis interactions^14,15^. Although AGs of *R. solani* are identified on the basis of hyphal anastomosis reactions, however reproducibility of these interactions needs experiences, is a time consuming process and can be affected by factors including laboratory environment, nutritional conditions and genetic stability^14,16^. Molecular approaches including DNA based sequence homology, restriction analysis of ribosomal DNA have been confirmed as reliable tools to differentiate isolates of *R. solani* into distinct clades corresponding to different AGs and subgroups^17,18^. Different anastomosis groups cause infection on differential hosts. AG-3 is most widely distributed in Pakistan which causes black scurf on potato^19^ however, it has also been reported to infect other solanaceous vegetable crops^20–22^. Other AGs including AG-2-1^22^, AG-2-2^23^, AG-4^24^ and AG-5^25^ have also been reported to infect tomato. Although, foot rot of tomato caused by *R. solani* has already been reported from Pakistan however, AG group composition of *R. solani* responsible for this infection is not determined. The objective of the present study is to determine anastomosis groups (AGs) of *R. solani* causing foot rot of tomato using tester isolates and restriction analysis of ribosomal DNA.

## Materials and Methods

### Sampling and isolation of *Rhizoctonia solani*

Surveillance of different tomato production areas of Pothohar region which includes districts; Jhelum, Chakwal, Attock, Rawalpindi, and Islamabad Capital Territory was done to collect diseased plants showing typical symptoms of foot rot and damping off. Pothohar region is situated between latitude 32.5°00′N to 34°00′N and altitude 72°00′E to 74°00′E in the Asian sub-continent with an elevation of 517 m above sea level and experiences semi-arid to humid climate^26^. Tomato is grown in scattered locations in the selected districts so purposive sampling was done. Disease prevalence and incidence percentage was calculated using formula;

$$\begin{array}{rcl}{\rm{Disease}}\,{\rm{prevalence}}\,( \% ) & = & \frac{{\rm{Locations}}\,{\rm{showing}}\,R.solani\,{\rm{infection}}}{{\rm{Total}}\,{\rm{number}}\,{\rm{of}}\,{\rm{locations}}\,{\rm{examined}}}\times 100\\ {\rm{Disease}}\,{\rm{incidence}}\,( \% ) & = & \frac{{\rm{No}}.\,{\rm{of}}\,{\rm{infected}}\,{\rm{plants}}}{{\rm{Total}}\,{\rm{number}}\,{\rm{of}}\,{\rm{plants}}\,{\rm{examined}}}\times 100\end{array}$$

A total of 117 symptomatic plant samples were collected. Sections of the stem (ca. 5 mm cube) were surface sterilized with 1% sodium hypochlorite solution, washed twice in tap water and blotted on sterilized filter paper were placed on 9 cm diameter Petri-plates of PDA incubated at 25 °C for 4 days. Hyphae resembling *Rhizoctonia*^27^ were identified under a microscope and pure cultures were obtained using the hyphal tipping technique. All isolates were maintained on Malt Extract Agar (MEA) and were preserved on barley grains by the method described by^28^.

### Cultural Characteristics and Microscopic studies of *R. solani*

Recovered isolates were initially identified as *Rhizoctonia* by culture characteristics on MEA as described by Sneh, *et al*.^29^. Hyphae of *Rhizoctonia* have right-angle branches, branches at the distal septae of cells and dolipore septa. Isolates were grown in 9 cm Petri plates containing 2% water agar (WA) at 25 °C for 4 days stained with 0.05% lactophenol blue and examined under a microscope to observe hyphal morphology. A number of nuclei per cell of *R. solani* were counted by staining hyphae with 1ug/ml of 4′-6 diamidino-2-phenylindole (DAPI stain). Petri plates were examined under a fluorescent microscope at 400X magnification to count a number of nuclei per cell.

### Pathogenicity Testing

Pathogenicity tests were performed according to the method described by Misawa and Kuninaga^22^. Plastic cell trays (53.49 cm L × 26.82 cm W) having 32 cells/ tray were filled with sterilized potting mixture i.e. sand: clay: farmyard manure at the rate of 1:1:1^30^. Four week old plants *cv*. Rio Grande were transplanted into the cells. The inoculum was prepared by colonizing isolates of *R. solani* on barley grains for 14 days. 10 g of ground barley grains colonized with each isolate of *R. solani* was mixed in the upper 2 cm layer of soil. Cells colonized only with ground barley grains were used as a control. Plants were grown at 25 ± 2 °C for 28 days. Infection on soil line level of the stem was categorized as −, no symptom; ±, brown lesion on part of the stem; +, brown lesion girdled the stem; ++, brown lesion girdled the stem and plants wilted. The trial was conducted with three replicates for 67 recovered isolates arranged in completely randomized design (CRD). The whole experiment was repeated twice.

### Anastomosis group typing

Generally, isolates of *R. solani* are identified based on hyphal interaction reactions, however, resolution of this method at subgroup level is insufficient^31–33^. Recovered isolates were subjected to Restriction fragment length polymorphism (RFLP) analysis of ribosomal DNA (rDNA) sequences. The results of AG group composition were further confirmed by hyphal anastomosis interactions.

### PCR–RFLP analysis

**Figure 1:**
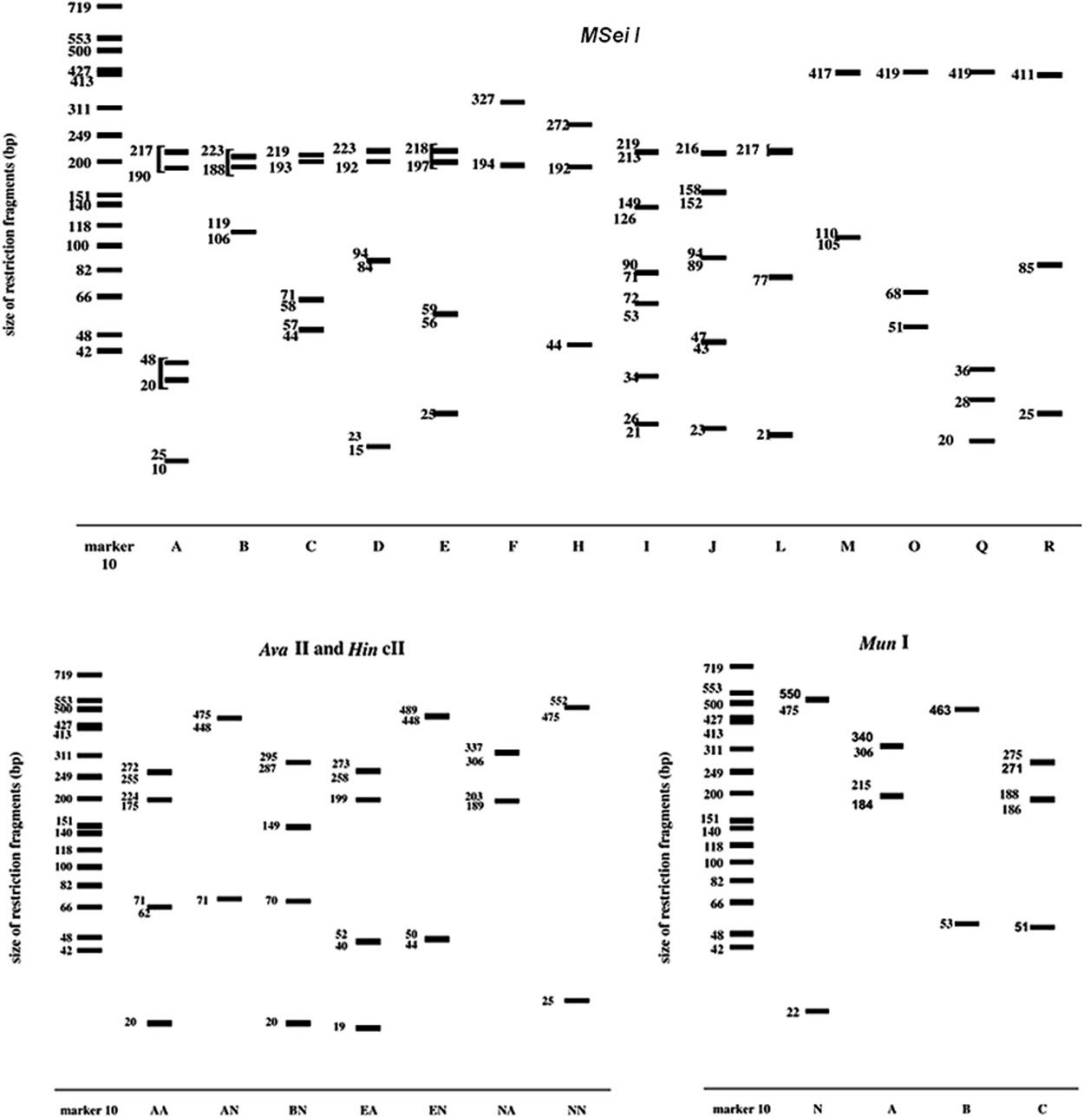
Restriction patterns revealed by RFLP analysis of internal transcribed spacers sequences of Rhizoctonia solani with discriminating enzymes^35^.

Isolates were maintained on malt extract broth (MEB) medium in 9 cm Petri plates incubated at 25 °C for 5 days. Mycelium mat for each isolate was harvested blot dried, lyophilized and ground to fine powder. DNA from each isolate was extracted using the standard protocol of Omniprep for fungi extraction kit (G-Biosciences) (Cat. # 786-399) and was subjected to PCR amplification of the ITS region with primers RS1 (5′-CCTGTGCACCTGTGAGACAG-3′) and RS4 (5′-TGTCCAAGTCAATGGACTAT-3′)^34^. PCR reaction mixture was prepared by adding 10 µL 5X Buffer, 1 µL dNTPs, 2.5 µL each forward and reverse primers, 1 µL MgCl_2_, 0.5 µL Taq Polymerase 2.5 µL DNA template in the final volume of 50 µL. A negative control (without DNA template) was always included in PCR reactions. Amplifications were performed in MJ Research Tetrad PTC-225 Thermal Cycler system (Bunker Lake Blvd. Ramsey, Minnesota, USA) with an initial denaturation at 94 °C for 2 min followed by 35 cycles of denaturation at 94 °C for 30 s, annealing at 55 °C for ITS for 30 s, extension at 72 °C for 1 min, and a final extension at 72 °C for 5 min. Aliquots of the PCR products were analyzed in 2% agarose gel stained with ethidium bromide and viewed using a UV transilluminator. Each PCR product was cleaned up using Sephadex G-50 and amplifications were further characterized using discriminating enzymes; *MseI*, *AvaII* + *HincII* and *MunI* as determined by Guillemaut, *et al*.^35^ (Fig. 1).

### Anastomosis interactions

Isolates were paired with tester strains of the respective AGs identified by PCR-RFLP. An agar disc (5 mm) was excised from the edge of the actively growing hyphae and placed on 1.5% WA coated clean glass slides having similar agar disc of tester strain of the known AG. After 48–72 hours when hyphae from each isolate overlapped, slides were stained with lactofuchsin and were examined under 400x magnification for hyphal anastomosis. Anastomosis reactions were classified from C0 to C3 where, C0 = no reaction, C1 = contact fusion, C2 = somatic fusion or perfect anastomosis and C3 = auto-anastomosis as described by Carling^36^. C3 type interactions; auto-anastomosis or self-pairing were used as positive control.

Twenty random locations on each glass slide were selected to observe hyphal interactions between unknown strain and the tester strain of respective AG and percentage fusion frequency (% FF) was determined as;

$$ \% \,FF=\frac{A\times 100}{B}$$

where^37,38^,

A = Sum of fusion locations (in C1, C2, C3) in 20 microscopic fields

B = Sum of contact points in 20 microscopic fields

Isolates pairing at more than 80% locations were confirmed as belonging to respective anastomosis group.

### Sequencing of ITS-5.8S rDNA and phylogeny

Molecular identification of the type isolates belonging to different AGs was accomplished by amplification of their ITS region using universal sense ITS1 (5′-TCCGTAGGTGAACCTGCGG-3′) and ITS4 (5′-TCCTCCGCTTATTGATATGC-3′) encoding ITS-1-5.8S-ITS-2^39^. PCR mixtures were prepared in a total volume of 50 µL containing 2.5 µL of the total DNA, 2.5 µL each forward and reverse primers, 10 µL 5X Buffer, 1 µL MgCl_2_, 1 µL dNTPs, and 0.5 µL of Taq DNA polymerase. A negative control (without DNA template) was always included in PCR reactions. PCR conditions were same as described in the PCR-RFLP analysis. The PCR amplified products were analyzed in 2% agarose gel (high resolution agarose, Q-BIOgen) in TAE buffer containing 40 mmol/L Tris–HCl (pH 7.9), 4 mmol/L sodium acetate, and 1 mmol/L EDTA (pH 7.9). Aliquots of the PCR products were analyzed in 2% agarose gel stained with ethidium bromide and viewed using a UV transilluminator. PCR products were purified using Sephadex G-50 and were sequenced in both directions (GenBank Accession; MG548644-48, MG844369-81) given in Table 1. The ITS sequence data was compared with those of related genera available in the National Center for Biotechnology Information (NCBI) GenBank. Sequence data was aligned using BioEdit software^40^ with Clustal W programme^41^. Phylogenetic and molecular evolutionary analyses were accomplished by constructing Maximum Likelihood tree with the ITS sequences for *R. solani* obtained from GenBank using Mega^42^ and MrBayes software^43^. The positions containing gaps and missing data were eliminated. Bootstrapping was performed at 1000 replications of the data being analyzed.

**Table 1:** *Rhizoctonia solani* isolates from tomato stem portions used in this study and Genbank accession number of their ITS regions.

Sr.	Isolate	Location	AG	Accession No
1	ATKT10	Attock	AG-2-1	MG844372
2	ISBT5	Islamabad	AG-2-1	MG844373
3	JHET4	Jhelum	AG-2-1	MG844374
4	RWPT14	Rawalpindi	AG-2-1	MG844375
5	ATKT17	Attock	AG-2-2	MG844376
6	CHKT8	Chakwal	AG-2-2	MG844377
7	RWPT8	Rawalpindi	AG-2-2	MG844378
8	ATKT9	Attock	AG-3-PT	MG548644
9	CHKT1	Chakwal	AG-3-PT	MG548645
10	CHKT5	Chakwal	AG-3-PT	MG548646
11	ISBT4	Islamabad	AG-3-PT	MG548647
12	JHET8	Jhelum	AG-3-PT	MG548648
13	RWPT5	Rawalpindi	AG-3-PT	MG844369
14	JHET11	Jhelum	AG-4-HGI	MG844370
15	RWPT4	Rawalpindi	AG-4-HGI	MG844371
16	ATKT6	Attock	AG-5	MG844379
17	CHKT4	Chakwal	AG-5	MG844380
18	JHET14	Jhelum	AG-5	MG844381

*Rhizoctonia solani* isolates from tomato used in this study and Genbank accession number of their ITS regions.

### Statistical analysis

Data pertaining to the pathogenicity testing was statistically analyzed using Genstat 6th edition^44^. Analysis of variance was used to test differences between variables and means were separated by means of Fisher’s protected least significant differences (LSD).

## Results

### Sampling and isolation of *Rhizoctonia solani*

*R. solani* infection was 100% prevalent to all the visited locations. District wise, maximum mean disease incidence was observed in Islamabad (38.7%) followed by district Attock (36.3%), district Rawalpindi (34.9%) and district Chakwal (29.6%) while minimum mean disease incidence was recorded in district Jhelum (27.5%). Lesions on infected plant stems were irregular in shape, water-soaked, brown in colour, and sunken in appearance. The disease was observed in patches of 6–12 plants. Wilting of diseased plants and stem damage with brown canker near soil line level of the stem was observed in mature plants, however, roots remained healthy. A total of 67 isolates of *R. solani* recovered on water agar (WA) medium started the hyphal growth from the second day of incubation. The hyphal tips of the actively growing mycelium were cultured on Malt Extract Agar (MEA) medium. The hyphal growth on MEA medium started on the second day, however, the growth was more vigorous than WA medium. Isolates incubated on MEA medium were light grey or medium to dark brown with abundant mycelial growths.

### Microscopic studies and morphology of *R. solani* isolates

**Figure 2:**
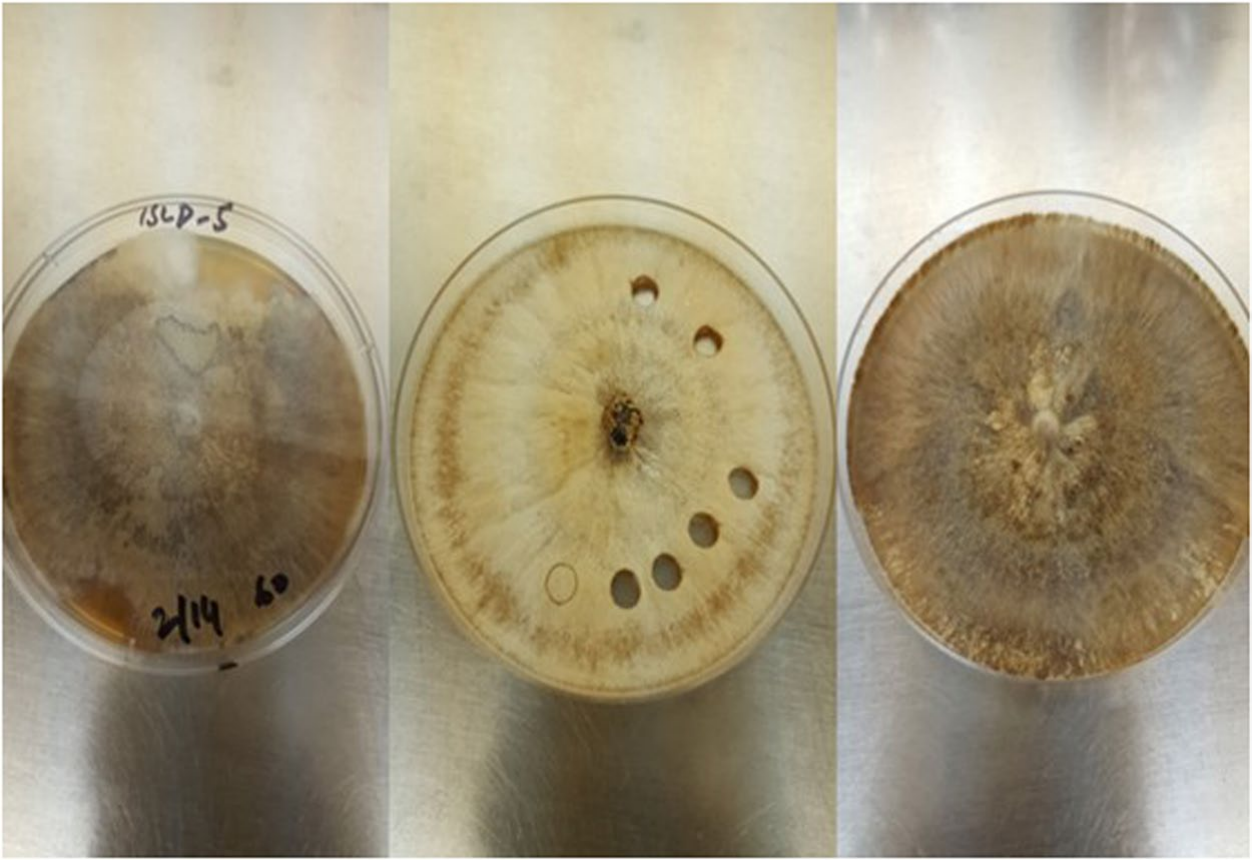
Colony morphology of *R. solani* isolates recovered from tomato.

**Figure 3:**
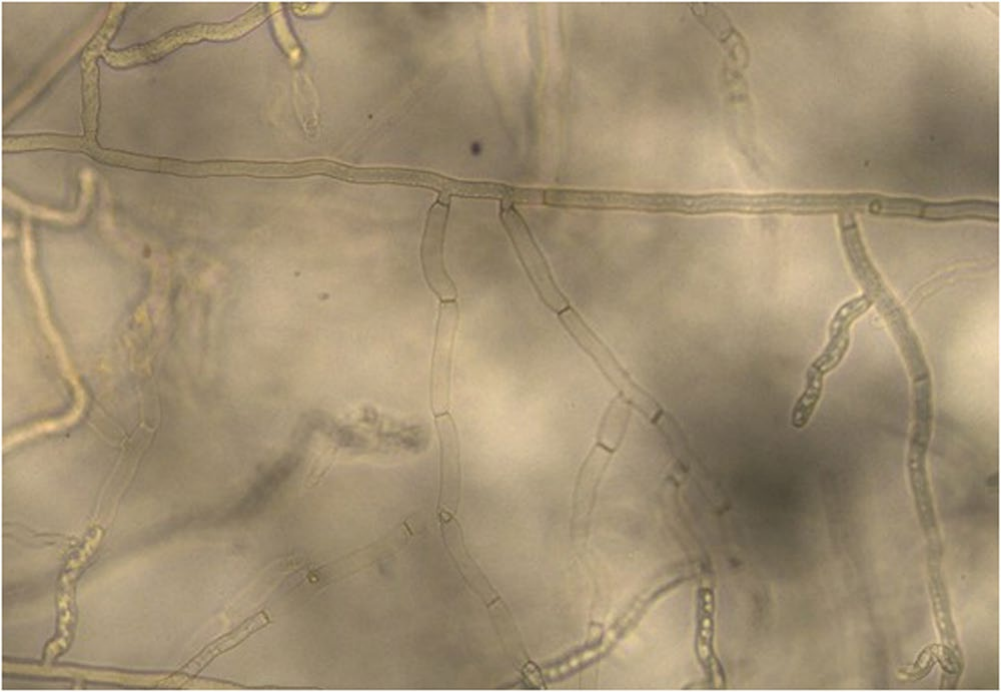
Branched hyphae of *R. solani* at right angle to each other with septum under light microscope.

**Figure 4:**
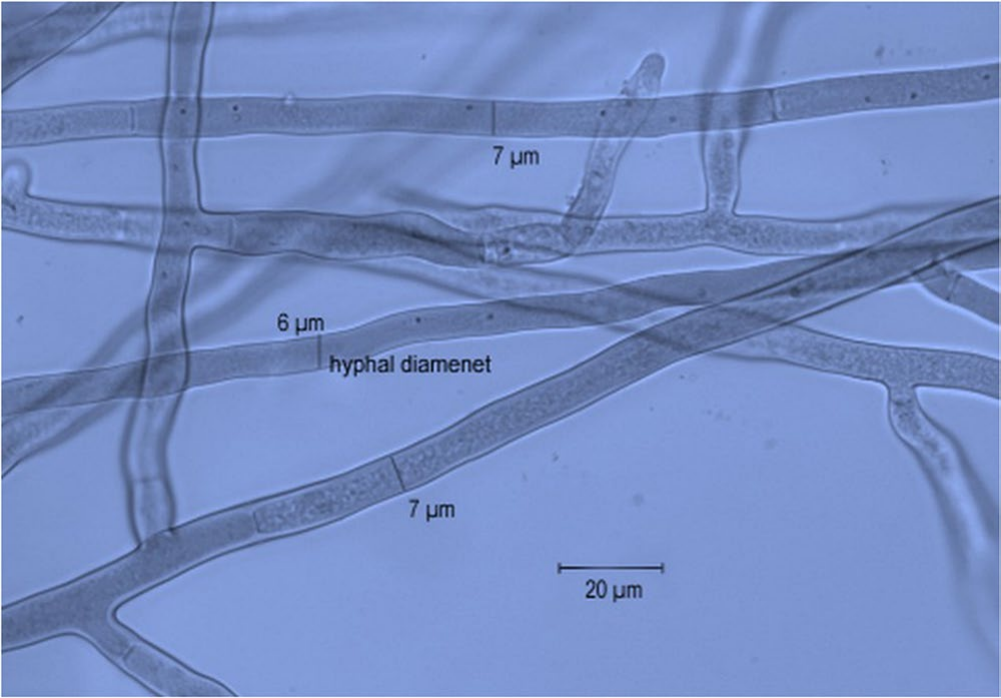
Hyphal diameter of *R. solani* isolates recovered from tomato.

**Figure 5:**
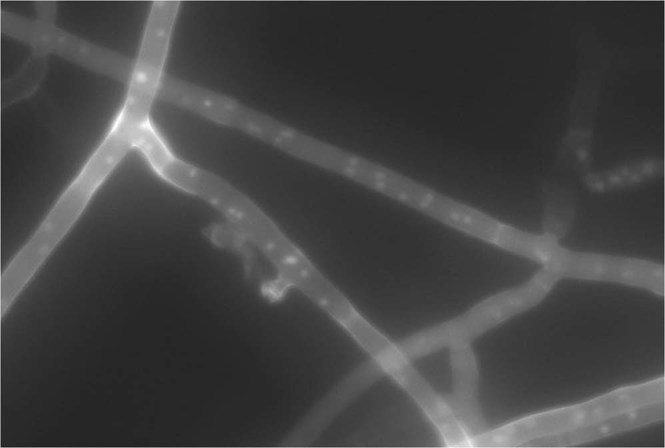
Nuclear number testing of *R. solani* isolates stained with DAPI (4′,6-diamidino-2-phenylindole).

Isolates were morphologically characterized according to the descriptions of *R. solani* by Ogoshi^37^ and Sneh, *et al*.^29^. With a considerable variation, all isolates exhibited typical *R. solani* colony and cultural characteristics (Fig. 2). Fungal hyphae were branched at right angles and a septum was always present in the branch of hyphae near the originating point with a slight constriction at the branch (Fig. 3). No conidia or conidiophores were observed. The hyphal distance between two septa varied from 67.6 to 149.8 μm (average 109.5 μm). The hyphal diameter of the isolates ranged between 5.1 to 8.1 μm (average 6.40 μm) as shown in Fig. 4. DAPI (4′-6 diamidino-2-phenylindole) stain was used to count a number of nuclei per cell of *R. solani*. Microscopic studies under a fluorescent light microscope revealed all isolates were multinucleate (Fig. 5). Seven days after incubation on MEA medium 89% of the isolates produced sclerotia, however, 11% of the isolates failed to produce sclerotia. The sclerotia developed from the middle to the edges of the colonies and were light to dark brown in the start and later turned dark brown to black in colour. The sclerotia were either rough or smooth. Most of the isolates produced rough sclerotia that were superficially available on the hyphal mass. Formation of the dark brown to black exudates was also observed in some of the recovered isolates. A detailed morphological description of the isolates is given in the table as Supplementary Information.

### Pathogenicity testing

All isolates varied in virulence to stem infections on tomato seedlings leading to wilting and/or plant death. Infection on soil line level of the stem was categorized as −, no symptom (avirulent); ±, brown lesion on part of the stem (Moderately virulent); +, brown lesion girdled the stem (Virulent); ++, brown lesion girdled the stem and plants wilted (Highly virulent). A wide variation in aggressiveness toward tomato was observed among *R. solani* isolates, as reflected in DI ranging from 0 to 51% for stem damage (Table 2). Out of sixty-seven isolates, 8% of the isolates showed an avirulent response, 10% showed moderately virulent, 19% showed virulent while 63% of the isolates showed a highly virulent response (Table 3).

**Table 2:** Disease index for *Rhizoctonia solani* isolates on Tomato (cv. Rio Grande).

Isolates	Disease Incidence	Isolates	Disease Incidence	Isolates	Disease Incidence
RWPT1	43 fg	CHKT9	35 ijk	JHET4	44 efg
RWPT2	45 def	CHKT10	18 op	JHET5	40 h
RWPT3	6 q	CHKT11	36 ijk	JHET6	22 mn
RWPT4	47 bcd	ATKT1	16 p	JHET7	48 bc
RWPT5	43 fg	ATKT2	43 fg	JHET8	46 cde
RWPT6	0 r	ATKT3	45 def	JHET9	0 r
RWPT7	51 a	ATKT4	0 r	JHET10	6 q
RWPT8	49 ab	ATKT5	23 mn	JHET11	37 i
RWPT9	22 mn	ATKT6	47 bcd	JHET12	46 cde
RWPT10	5 q	ATKT7	43 fg	JHET13	5 q
RWPT11	40 h	ATKT8	23 m	JHET14	43 fg
RWPT12	49 ab	ATKT9	51 a	JHET15	40 h
RWPT13	28 l	ATKT10	47 bcd	JHET16	0 r
RWPT14	46 cde	ATKT11	43 fg	JHET17	48 bc
RWPT15	0 r	ATKT12	6 q	JHET18	22 mn
CHKT1	33 k	ATKT13	51 a	JHET19	46 cde
CHKT2	35 ijk	ATKT14	49 ab	ISBT1	7 q
CHKT3	22 mn	ATKT15	23 m	ISBT2	37 i
CHKT4	35 ijk	ATKT16	40 h	ISBT3	23 mn
CHKT5	36 ij	ATKT17	49 ab	ISBT4	46 cde
CHKT6	22 mn	JHET1	46 cde	ISBT5	43 fg
CHKT7	7 q	JHET2	42 gh		
CHKT8	34 jk	JHET3	21 no

**Table 3:** Pathogenicity determination of sixty-seven *R. solani* isolates on Tomato (cv. Rio Grande).

Avirulent	RWPT6, RWPT15, ATKT4, JHET9, JHET16
Moderately virulent	RWPT3, RWPT10, CHKT7, ATKT12, JHET10, JHET13, ISBT1
Virulent	RWPT9, RWPT13, CHKT3, CHKT6, CHKT10, ATKT1, ATKT5, ATKT8, ATKT15, JHET3, JHET6, JHET18, ISBT3
Highly virulent	RWPT1, RWPT2, RWPT4, RWPT5, RWPT7, RWPT8, RWPT11, RWPT12, RWPT14, CHKT1, CHKT2, CHKT4, CHKT5, CHKT8, CHKT9, CHKT11, ATKT2, ATKT3, ATKT6, ATKT7, ATKT9, ATKT10, ATKT11, ATKT13, ATKT14, ATKT16, ATKT17, JHET1, JHET2, JHET4, JHET5, JHET7, JHET8, JHET11, JHET12, JHET14, JHET15, JHET17, JHET19, ISBT2, ISBT4, ISBT5

### PCR–RFLP analysis

**Figure 6:**
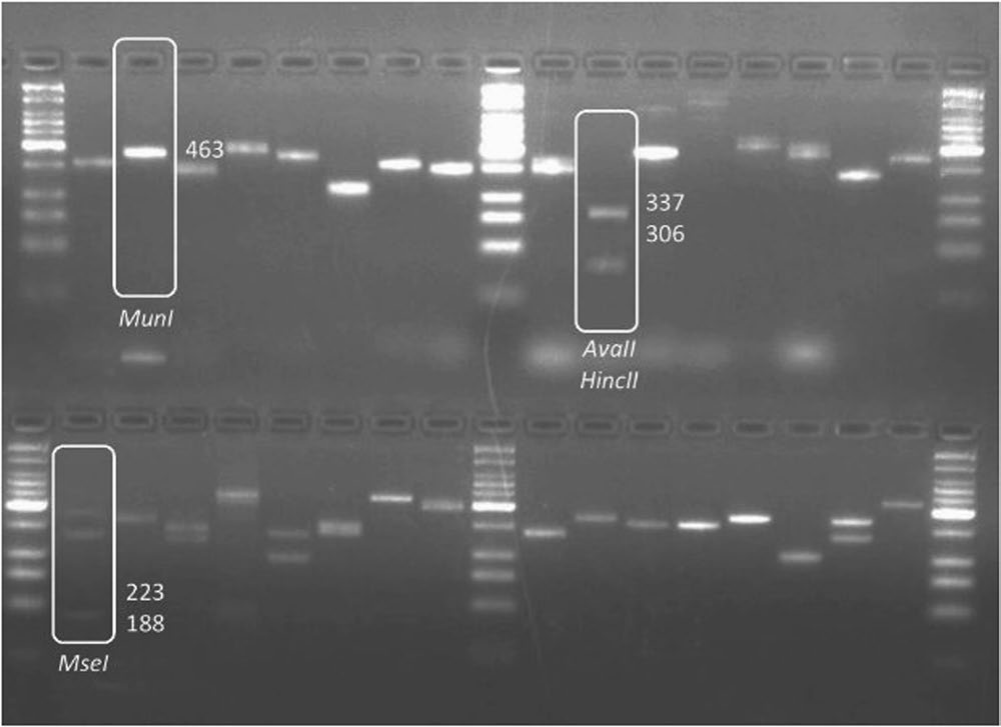
PCR-RFLP restriction patterns revealed by discriminating enzymes (*MseI, AvaII* + *HincII* and *MunI*).

Discriminating enzymes (*MseI*, *AvaII* + *HincII* and *MunI*) restricted each fragment to multiple locations for each marker as shown in Fig. 6. The combination of these markers was used to designate specific anastomosis group to each isolate (Table 4). A total of twenty-seven isolates were assigned AG-3 PT as they shared BNAN and FNAN RFLP type. Twenty-three isolates shared BNAN and four isolates shared FNAN RFLP type. *MseI* restricted the fragments at two locations; 106–119, 188–233 bp for marker B and 194, 327 bp for marker F. The restriction patterns for combination of *AvaII* and *HincII* were 189–203 and 306–337 bp for marker NA, while the restriction patterns corresponding to MunI were 22 and 475–550 bp for marker N. This indicates majority of the recovered isolates belong to AG-3 PT as expected strains for crop types.

**Table 4:** Anastomosis groups (AGs) assigned using PCR-RFLP and hyphal anastomosis interactions.

Isolate	Targeted restriction patterns				Anastomosis Group	Hyphal Interaction
	*MseI*	*AvaII*	*HincII*	*MunI*		
RWPT1	B	N	A	N	AG-3-PT	AG-3
RWPT2	B	N	A	N	AG-3-PT	AG-3
RWPT4	I	E	A	A	AG-4-HGI	AG-4
RWPT5	F	N	A	N	AG-3-PT	AG-3
RWPT7	B	N	A	N	AG-3-PT	AG-3
RWPT8	B	A	A	N	AG-2-2	AG-2-2
RWPT11	B	N	A	N	AG-3-PT	AG-3
RWPT12	B	N	A	N	AG-3-PT	AG-3
RWPT14	D	A	N	A	AG-2-1	Unknown
JHET1	B	N	A	N	AG-3-PT	AG-3
JHET2	B	N	A	N	AG-3-PT	AG-3
JHET4	B	B	N	A	AG-2-1	AG-2-1
JHET5	B	N	A	N	AG-3-PT	AG-3
JHET7	B	N	A	N	AG-3-PT	AG-3
JHET8	B	N	A	N	AG-3-PT	AG-3
JHET11	I	E	A	A	AG-4-HGI	AG-4
JHET12	B	N	A	N	AG-3-PT	AG-3
JHET14	H	A	A	C	AG-5	AG-5
JHET15	B	B	N	A	AG-2-1	Unknown
JHET17	B	N	A	N	AG-3-PT	AG-3
JHET19	B	N	A	N	AG-3-PT	AG-3
ATKT2	B	N	A	N	AG-3-PT	AG-3
ATKT3	F	N	A	N	AG-3-PT	AG-3
ATKT6	H	A	A	C	AG-5	AG-5
ATKT7	F	N	A	N	AG-3-PT	AG-3
ATKT9	B	N	A	N	AG-3-PT	AG-3
ATKT10	B	B	N	A	AG-2-1	AG-2-1
ATKT11	B	N	A	N	AG-3-PT	AG-3
ATKT13	B	A	A	N	AG-2-2	AG-2-2
ATKT14	B	N	A	N	AG-3-PT	AG-3
ATKT16	F	N	A	N	AG-3-PT	AG-3
ATKT17	B	A	A	N	AG-2-2	AG-2-2
CHKT1	B	N	A	N	AG-3-PT	AG-3
CHKT2	B	N	A	N	AG-3-PT	AG-3
CHKT4	H	A	A	C	AG-5	AG-5
CHKT5	B	N	A	N	AG-3-PT	AG-3
CHKT8	B	A	A	N	AG-2-2	AG-2-2
CHKT9	B	N	A	N	AG-3-PT	AG-3
CHKT11	D	A	N	A	AG-2-1	AG-2-1
ISBT2	B	N	A	N	AG-3-PT	AG-3
ISBT4	B	N	A	N	AG-3-PT	AG-3
ISBT5	B	B	N	A	AG-2-1	AG-2-1

Six isolates were assigned AG-2-1 and four isolates to AG-2-2 based on the restriction patterns. The restriction patterns corresponding to *MseI* were 106–109 and 188–233 bp for marker B, 44–57, 58–71 and 193–219 for marker C while 15–23, 84–94 and 192–233 for marker D. The restriction patterns for combination of *AvaII* and *HincII* were 20, 63–71, 175–224 and 255–272 bp for marker AA, 71, 448–475 bp for marker AN while 20 b, 70 b, 149 and 287–295 bp for marker BN. *MunI* restricted the fragments at two locations; 184–215, 306–340 bp for marker A and 22,475–550 bp for marker N. Out of six isolates belonging to AG-2-1, four isolates shared BBNA while two isolates shared DANA RFLP type. Isolates assigned AG-2-2 shared BAAN RFLP type.

Three isolates belong to AG-5 as they shared HAAC types. The restriction patterns corresponding to *MseI* were 44, 192 and 272 bp for marker H. The restriction patterns for a combination of *AvaII* and *HincII* were 20, 63–71, 175–224 and 255–272 bp for marker AA. *MunI* restricted the fragments at two locations; 51, 186–188, 271–275 bp for marker C.

Two isolates were designated as AG-4 HGI based on the RFLP type; IEAA they shared. The restriction patterns corresponding to *MseI* were 21–26, 34, 53–72, 71–90, 126–149 and 213–219 bp for marker I while patterns conforming combination of *AvaII* and *HincII* were 19, 40–50, 199 and 258–273 for marker EA. *MunI* restricted the fragments at two locations; 184–215, 306–340 bp for marker A.

### Hyphal Anastomosis Interaction

A considerable variation in the hyphal interactions as; C0 (no reaction), C1 (only contact fusion), C2 (somatic fusion or perfect anastomosis) and C3 (auto anastomosis) were observed. A total of twenty-seven isolates formed C2 = somatic fusion of perfect anastomosis interactions with the tester strain AG-3. C3 type or self anastomosis interactions were not taken into consideration as they represented the interactions between hyphae of the same isolates. The C2 type hyphal fusion frequency among these isolates was more than 80%. Selected isolates were identified as members of AG-3. Eleven isolates showed perfect fusion with the tester strain of AG-2. Five isolates showed strong somatic fusion with the tester strains of AG-5 while two isolates fused with the tester strain of AG-4. All these isolates confirmed the identity of PCR-RFLP analysis (Table 4). Two isolates RWPT-14 and JHET15 showed somatic fusion with the tester strains of AG-3, AG-2, AG-4 and AG-6 at 45–69% fusion frequency. The identity of these two isolates was subjected to molecular characterization.

### Sequencing of ITS-5.8S rDNA and phylogeny

DNA fragments of eighteen type isolates representing anastomosis groups identified by PCR-RFLP and hyphal anastomosis interactions together with two isolates showing hyphal interactions with more than one tester strain *R. solani* AGs were subjected to PCR amplification with a set of universal sense primers; ITS1 and ITS4 encoding ITS-1-5.8S-ITS-2^39^. The amplicons generated a fragment of approximately 700 bp on an agarose gel. The ITS region (ITS1, 5.8S rDNA, and ITS2) of each isolate was sequenced in both sense and antisense directions. BLAST analysis of these sequences with the known sequences of *R. solani* AGs from NCBI GenBank confirmed the identity of respective AGs (99–100% sequence identities) previously revealed by PCR-RFLP and hyphal interactions.

**Figure 7:**
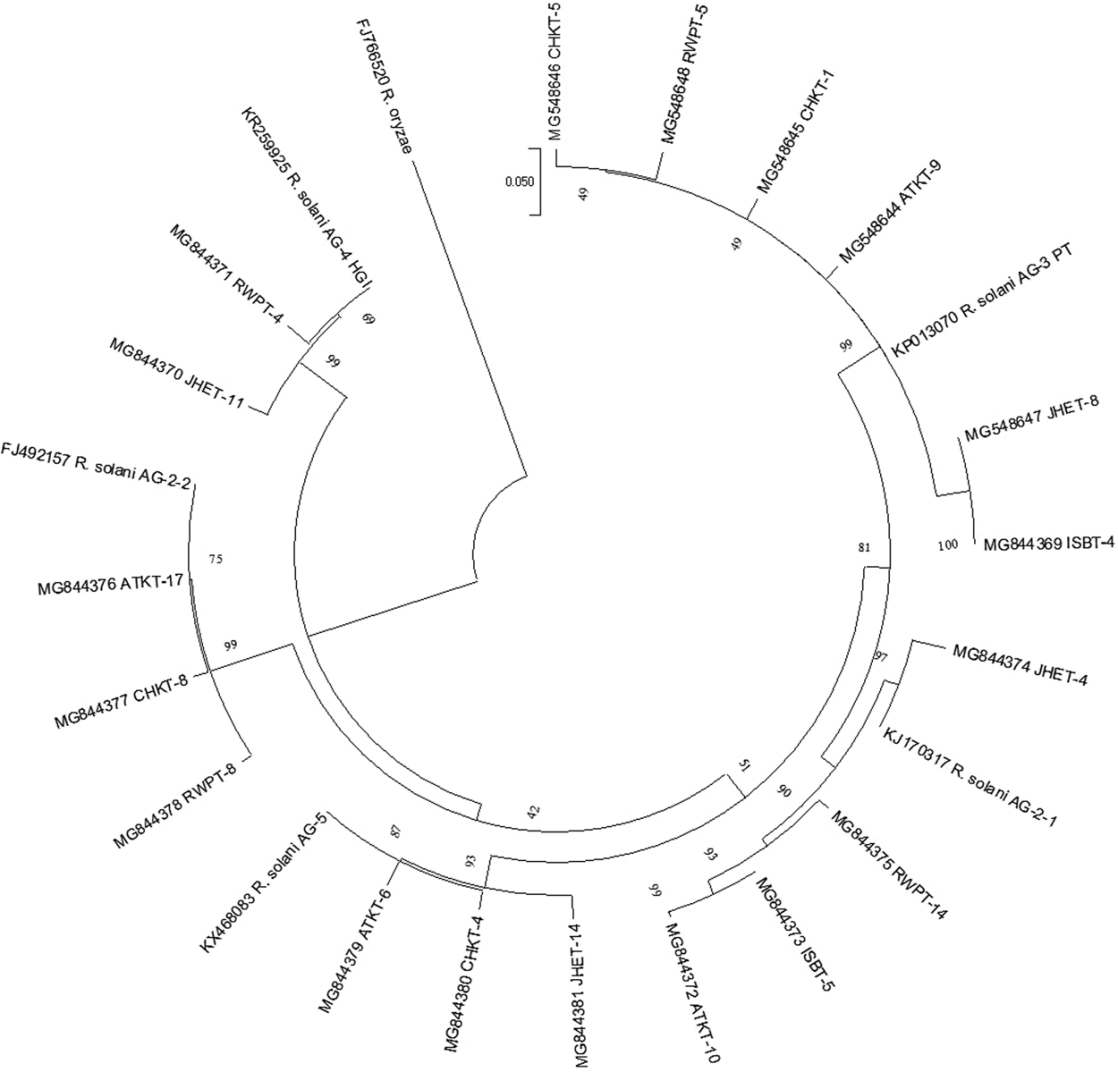
Phylogenetic analysis of *Rhizoctonia solani* isolates infecting tomato.

DNA sequences of the isolates representing AG-3 PT, AG-2-1, AG-5, AG-2-2 and AG-4 HGI formed different clades with 99, 90, 93, 99 and 99% bootstrap support respectively (Fig. 7). The percentage of trees in which the associated taxa clustered together is shown next to the branches. Initial tree(s) for the heuristic search were obtained automatically by applying Neighbor-Join and BioNJ algorithms to a matrix of pairwise distances estimated using the Maximum Composite Likelihood (MCL) approach and then selecting the topology with superior log likelihood value. Each tree is drawn to scale, with branch lengths measured in the number of substitutions per site (next to the branches). The analysis involved 24 nucleotide sequences. Codon positions included were 1st + 2nd + 3rd + Noncoding. All positions containing gaps and missing data were eliminated. There was a total of 514 positions in the final dataset. Evolutionary analyses were conducted in MEGA7^45^.

Isolates; CHKT5, RWPT, CHKT1, ATKT9, JHET8 and ISBT4 shared the clade of reference isolate AG-3 PT. Within this clade, two isolates; CHKT5 and RWPT5 made a distinct cluster with a bootstrap support of 49% from all other isolates. Isolate JHET4 formed a different cluster at 97% bootstrap value from isolates; ATKT10, RWPT14, ISBT5 while all shared the clade of reference isolate of AG-2-1. Isolates; JHET14, CHKT4 and ATKT6 formed a clade with reference isolate of AG-5 while isolate CHKT4 formed different cluster from other isolates with 93% bootstrap support within this clade. Isolates; RWPT8, CHKT8 and ATKT17 shared the same clade with reference isolate AG-2-2.

## Discussion

Since the climate of Pothohar region has considerable temperature and moisture variations including semiarid and sub-humid regions^46^, *R. solani* can survive under both cool and warm soils. It can remain active at a range of temperatures^47^, and is well adapted to survive unfavourable conditions as it remains dormant as sclerotia^48^. The optimum temperature ranges 24–31 °C for vegetative growth and the emergence of tomato seedlings also provide optimum temperatures for *R. solani* disease development; 24–32 °C^49^. Pothohar region receives an average of 1,249 mm rainfall of which more than 65% is received in monsoon. Soil moisture greatly influences the amount of *R. solani* inoculum in the soil^50^ that ultimately favour the disease development^30,51,52^. Multicropping and the intercropping are the common practices adopted by the farmers of the region. The use of noncertified seeds is also commonly practised as the same germplasm of the few local varieties is used for cultivation year after years. The most commonly used tomato varieties are Money Maker, Roma Rio Grande and Tropic^53^. In present studies, Rio Grande was the most susceptible to *R. solani* infection. It is well accepted that the occurrence of soil-borne pathogens including *R. solani* is greatly influenced by intensive cropping^54–56^. Solanaceous vegetable cultivation on the same fields also helps in the inoculum multiplication, however, this pathogen is also well adapted for life outside the host plants^47,57^. A substantial variation in disease incidence among different locations may be attributed to prevailing environmental conditions and different levels of susceptibility in the growing cultivars.

In the present study, a total of 67 isolates of *R. solani* were recovered from diseased tomato plant samples on malt extract agar (MEA) medium. With considerable variations, all recovered isolates exhibit typical *R. solani* colony and cultural characteristics. All isolates were multinucleate with 3–8 nuclei per cell and had hyphal branching at a right angle to the constriction found at the point of branching mycelium, a known feature for *R. solani* described by Sneh, *et al*.^29^. A septum, that is of immense taxonomical importance was always present near the branching junction. All 67 isolates were morphologically differentiated and classified on the basis of septal distance, hyphal diameter, no. of sclerotia, texture and topography of sclerotia.

Morphological variations between isolates from different geographical regions have previously been studied by Parmeter, *et al*.^58^, Sharma, *et al*.^59^ and Goswami, *et al*.^60^. Sunder, *et al*.^61^ reported colony colour ranged from brown, light brown, dark brown and yellowish brown. Neeraja, *et al*.^62^ and Vineeta, *et al*.^63^ reported the significant importance of the mycelial and sclerotial characteristics in categorizing *R. solani* isolates into distinct groups. The septal distance ranged between 67.6 to 149.8 μm. The hyphal diameter ranged between 5.1 to 8.1 μm. Hansen^64^ also found that hyphal diameter ranged from 4.3–8.0. These findings were also in line with the findings of Vijayan and Nair^65^. Meyer, *et al*.^66^ found some *R. solani* isolates may not produce sclerotia under certain cultural conditions. Therefore, the absence of sclerotia may not be criteria for the mycelium to be excluded from *R. solani*. Location of the sclerotial production as superficial or immersed was also supported by the findings of Vineeta, *et al*.^63^. Anderson^67^ and Hoa^68^ also differentiated sclerotia from different isolates on the basis of colour. The findings of Sinha and Ghufran^69^ also supported the variations in colony colour, number size and the colour of sclerotia formed. Variations in cultural characteristics were in line with Kuiry, *et al*.^70^. Categorizing isolates based on the cultural and morphological features showed the diversity among the isolates was not correlated with their origin of the collection as supported by Baird, *et al*.^71^. The morphological classes based on the present studies were however, conservative since only MEA medium was used for this study.

The isolates were subjected to pathogenicity determination under greenhouse experiments on tomato. Distinct variations were observed in the pathogenicity of these isolates. The pathogenicity results showed that isolates within the same AG had variability in pathogenicity and virulence, which may be isolate dependent rather than AG dependent. Among sixty-seven isolates tested for pathogenicity on tomato *cv*. Rio Grande 63% of the isolates showed a highly virulent response. There was no correlation between mycelial growth and virulence. This was supported by the results of Basu *et al*. (2004).

Isolates purified using hyphal tipping on PDA medium were preserved by colonizing on hulled barley grains maintained at 4 °C. Sneh, *et al*.^72^ and Webb, *et al*.^73^ also used the cryogenic storage method for long-term preservation of *R. solani* isolates.

Hyphal anastomosis interactions are considered to be a more accurate method for accommodating isolates of *R. solani* into AGs however, reproducibility of this method for a large number of populations is difficult and its reliability at subgroup identification is unsatisfactory^17,32^. A total of 42 highly virulent isolates were subjected to PCR-RFLP analysis with four discriminating enzymes (*MseI*, *AvaII* + *HincII* and *MunI*) to categorize them into different at AGs. Results of the PCR-RFLP analysis revealed 27 isolates belonged to AG-3 PT while 6, 4, 3 and 2 isolates belonged to AG-2-1, AG-2-2, AG-5 and AG-4 HGI respectively.

Each isolate was paired with the tester strain of respective AG identified by PCR-RFLP. Environmental factors including temperature variations and nutritional stress may greatly influence the vegetative compatibility of the isolates^74^. In present studies, compatibility of the isolates with tester strains was tested on MEA medium with optimum growth conditions. Among four types of anastomosis reactions; C0 to C3 only C2 reactions were considered as somatic fusion or perfect anastomosis as described by Carling^36^. All isolates except two confirmed the AG group identity revealed by PCR-RFLP while the identity of two isolates was not confirmed as they produced hyphal interactions with more than one tester strains of the known AGs. The identity of these two isolates was unknown and they were further subjected to sequence analysis of their ITS-5.8S rDNA.

Type isolates representing anastomosis groups identified by PCR-RFLP and confirmed by hyphal anastomosis interactions together with two unknown isolates were subjected to PCR amplification with a set of universal sense primers; ITS1 and ITS4 encoding ITS-1-5.8S-ITS-2. BLAST analysis of the obtained sequences with the known sequences of *R. solani* AGs from NCBI GenBank confirmed the identity of respective AGs (99–100% sequence identities). All the isolates showed heterogeneity in their ITS sequences. Sequence analysis also confirmed the identity of unknown isolates. DNA sequences of *R. solani* isolates from Pakistan infecting tomato crop are now available at NCBI.

AG group composition of the virulent isolates revealed 64.2% isolated belonged to AG-3 PT and they were predominant to all tomato production areas of Pothohar region concordant with the previous findings by Rauf, *et al*.^19^ on potato. The frequency of the other AGs was far less than AG-3 as AG-2-1 14.2%, AG-2-2 9.5%, AG-5 7.1% and AG-4 HGI 4.7%. The association of AG-3 PT and AG-5 with foot rot of tomato was also supported by the findings of Muzhinji, *et al*.^32^ however the association of AG-2-1 was in contradiction. AG-2-1 and AG-2-2 have also been reported to cause foot rot of tomato^22^. The relative proportion of AG-3 PT, AG-5 and AG-2-1 in the present study is in line with the findings of Campion, *et al*.^75^ and Chand and Logan^76^. Majority of the AG-2-1, AG-2-2 and AG-4 HGI isolates were found in the three districts; Attock, Rawalpindi, and Jhelum. The localized occurrence of these two AGs could be attributed to the susceptible preceding crops like potato and chilli.

## Conclusion and Recommendations

Study reports status of *R. solani* infection with reference to Pakistan along with the occurrence of five anastomosis groups of *R. solani* on tomato with various levels of intensities. AG-3 PT was found to be the most prevalent and aggressive compared to other AGs in all tomato growing areas. Stringent surveillance for the occurrence of *R. solani* AGs on vegetable crops is needed. While evolving new varieties of tomato, *R. solani* isolates belonging to the reported anastomosis groups (AGs) may be used in breeding program.

## Supplementary information

Supplementary Data Table
