# Supplementary material for: Anastomosis Groups of Rhizoctonia solani associated with tomato foot rot in Pothohar Region of Pakistan
Source: Sci Rep. 2019 Mar 7;9:3910. doi: 10.1038/s41598-019-40043-5 (PMC6405938; doi:10.1038/s41598-019-40043-5)
Supplement: Supplementary file 1 — Supplementary Data Table [file 41598_2019_40043_MOESM1_ESM.docx]

**Anastomosis Groups of *Rhizoctonia solani* associated with tomato foot rot in Pothohar Region of Pakistan**

**Amjad Shahzad Gondal, Abdul Rauf, Farah Naz**

*Department of Plant Pathology, PMAS Arid Agriculture University Rawalpindi, Pakistan.*

Email: amjadshahzad@live.com

Tel: +1 724 990 0670

Table: Morphological characterization of sixty-seven isolates of *Rhizoctonia solani* recovered from portions of diseased tomato samples collected from Potohar region during 2014 and 2015 crop season.

| Isolate | Colony Color | Constriction | Colony Diameter cm | Hyphal Length  µm | Hyphal Width  µm | Nuclear Condition | Sclerotia  Color | Sclerotia Topography | Sclerotia Number | Sclerotia Texture | |
| --- | --- | --- | --- | --- | --- | --- | --- | --- | --- | --- | --- |
| RWPT1 | Medium brown | Present | 7.6 | 69.1 | 6.1 | Multinucleate | Medium to dark brown | Immersed | 51 | Rough | |
| RWPT2 | Dark brown | Present | 7.9 | 145.7 | 6.7 | Multinucleate | Dark brown | Superficial | 38 | Rough | |
| RWPT3 | Dark brown | Present | 7.3 | 96.4 | 5.9 | Multinucleate | Dark brown | Immersed | 31 | Rough | |
| RWPT4 | Dark brown | Present | 8 | 99.4 | 6.7 | Multinucleate | Medium to dark brown | Immersed | 42 | Rough | |
| RWPT5 | Medium brown | Present | 7.6 | 101.8 | 6.4 | Multinucleate | Medium to dark brown | Superficial | 37 | Smooth | |
| RWPT6 | Medium brown | Present | 7.1 | 108 | 6.1 | Multinucleate | Medium to dark brown | Superficial | 27 | Rough | |
| RWPT7 | Hyaline to light brown | Present | 8.3 | 67.6 | 5.3 | Multinucleate | Dark brown | Immersed | 47 | Smooth | |
| RWPT8 | Dark brown | Present | 7.7 | 122.6 | 7.2 | Multinucleate | Light brown to dark brown | Superficial | 38 | Rough | |
| RWPT9 | Dark brown | Present | 8.3 | 146.9 | 7.2 | Multinucleate | Medium to dark brown | Superficial | 32 | Rough | |
| RWPT10 | Hyaline to light brown | Present | 8.2 | 149.2 | 7.2 | Multinucleate | Medium to dark brown | Superficial | 31 | Smooth | |
| RWPT11 | Medium brown | Present | 7.2 | 96.2 | 6.4 | Multinucleate | Medium to dark brown | Superficial | 45 | Rough | |
| RWPT12 | Hyaline to light brown | Present | 7.1 | 148.7 | 7.5 | Multinucleate | Medium to dark brown | Superficial | 31 | Smooth | |
| RWPT13 | Hyaline to light brown | Present | 8.1 | 70.9 | 5.9 | Multinucleate | Medium to dark brown | Superficial | 26 | Smooth | |
| RWPT14 | Dark brown | Present | 7.7 | 99.9 | 6.8 | Multinucleate | Medium to dark brown | Superficial | 42 | Rough | |
| RWPT15 | Hyaline to light brown | Present | 7.8 | 107.2 | 5.9 | Multinucleate | Light brown to dark brown | Superficial | 51 | Rough | |
| CHKT1 | Hyaline to light brown | Present | 8.2 | 71.4 | 5.3 | Multinucleate | Dark brown | Immersed | 42 | Rough | |
| CHKT2 | Dark brown | Present | 7.3 | 149.8 | 7.8 | Multinucleate | Dark brown | Superficial | 39 | Smooth | |
| CHKT3 | Dark brown | Present | 7.6 | 119.1 | 6.8 | Multinucleate | White to light brown | Superficial | 39 | Rough | |
| CHKT4 | Hyaline to light brown | Present | 7.6 | 114.9 | 6.4 | Multinucleate | - | Not present | 0 | Not present | |
| CHKT5 | Medium brown | Present | 6.9 | 97 | 6.7 | Multinucleate | Medium to dark brown | Superficial | 45 | Rough | |
| CHKT6 | Dark brown | Present | 8.3 | 117.7 | 6.9 | Multinucleate | Medium to dark brown | Superficial | 37 | Rough | |
| CHKT7 | Hyaline to light brown | Present | 7.7 | 99.8 | 6.2 | Multinucleate | Dark brown | Superficial | 40 | Smooth | |
| CHKT8 | Hyaline to light brown | Present | 7.8 | 105.9 | 7.7 | Multinucleate | Light brown to dark brown | Immersed | 45 | Rough | |
| CHKT9 | Hyaline to light brown | Present | 8.2 | 117.2 | 6.6 | Multinucleate | Medium to dark brown | Superficial | 32 | Smooth | |
| CHKT10 | Dark brown | Present | 7.2 | 111.1 | 7.1 | Multinucleate | Light brown to dark brown | Superficial | 29 | Rough | |
| CHKT11 | Hyaline to light brown | Present | 7.1 | 74.5 | 5.4 | Multinucleate | - | Not present | 0 | Not present | |
| ATKT1 | Dark brown | Present | 7.8 | 71.9 | 5.8 | Multinucleate | - | Not present | 0 | Not present | |
| ATKT2 | Dark brown | Present | 7.8 | 146.3 | 7.2 | Multinucleate | Medium to dark brown | Superficial | 32 | Rough | |
| ATKT3 | Medium brown | Present | 8.3 | 103 | 5.2 | Multinucleate | Medium to dark brown | Superficial | 39 | Rough | |
| ATKT4 | Dark brown | Present | 7.7 | 118.2 | 6.9 | Multinucleate | Medium to dark brown | Immersed | 37 | Rough | |
| ATKT5 | Hyaline to light brown | Present | 7.8 | 136.8 | 7.3 | Multinucleate | Brown | Superficial | 50 | Rough | |
| ATKT6 | Dark brown | Present | 7.2 | 148.5 | 8.1 | Multinucleate | - | Not present | 0 | Not present | |
| ATKT7 | Medium brown | Present | 7.3 | 89.6 | 6.2 | Multinucleate | Light brown to dark brown | Superficial | 44 | Rough | |
| ATKT8 | Medium brown | Present | 7.8 | 122.1 | 5.4 | Multinucleate | Light brown to dark brown | Superficial | 39 | Rough | |
| ATKT9 | Medium brown | Present | 6.9 | 121.2 | 5.9 | Multinucleate | Light brown to dark brown | Superficial | 39 | Rough | |
| ATKT10 | Medium brown | Present | 8.3 | 99.7 | 5.4 | Multinucleate | Dark brown | Immersed | 48 | Rough | |
| ATKT11 | Dark brown | Present | 7.8 | 106.4 | 6 | Multinucleate | Medium to dark brown | Immersed | 24 | Smooth | |
| ATKT12 | Hyaline to light brown | Present | 6.8 | 78.9 | 5.5 | Multinucleate | Dark brown | Superficial | 45 | Rough | |
| ATKT13 | Dark brown | Present | 7.7 | 113.1 | 6.5 | Multinucleate | - | Not present | 0 | Not present | |
| ATKT14 | Dark brown | Present | 7.7 | 99.9 | 6.8 | Multinucleate | Medium to dark brown | Superficial | 42 | Rough | |
| ATKT15 | Medium brown | Present | 7.8 | 114.3 | 6.8 | Multinucleate | Brown | Immersed | 25 | Rough | |
| ATKT16 | Dark brown | Present | 7.7 | 101.2 | 6.5 | Multinucleate | Light brown to dark brown | Superficial | 50 | Rough |  |
| ATKT17 | Hyaline to light brown | Present | 8.2 | 117.2 | 6.6 | Multinucleate | Medium to dark brown | Superficial | 32 | Smooth | |
| JHET1 | Dark brown | Present | 7.7 | 136.2 | 6.5 | Multinucleate | White to light brown | Superficial | 31 | Rough | |
| JHET2 | Dark brown | Present | 7.8 | 99.3 | 6.2 | Multinucleate | Medium to dark brown | Superficial | 26 | Rough | |
| JHET3 | Hyaline to light brown | Present | 7.7 | 129.4 | 6.2 | Multinucleate | Medium to dark brown | Immersed | 42 | Smooth | |
| JHET4 | Dark brown | Present | 7.8 | 100.9 | 5.8 | Multinucleate | Dark brown | Immersed | 27 | Smooth | |
| JHET5 | Dark brown | Present | 8.2 | 94.5 | 6.7 | Multinucleate | Dark brown | Superficial | 32 | Rough | |
| JHET6 | Dark brown | Present | 7.2 | 89.5 | 5.5 | Multinucleate | Light brown to dark brown | Superficial | 45 | Smooth | |
| JHET7 | Medium brown | Present | 7.3 | 137.2 | 7.2 | Multinucleate | Brown | Superficial | 39 | Rough | |
| JHET8 | Dark brown | Present | 7.8 | 114 | 5.1 | Multinucleate | Medium to dark brown | Superficial | 31 | Rough | |
| JHET9 | Dark brown | Present | 6.8 | 76.7 | 5.7 | Multinucleate | - | Not present | 0 | Not present | |
| JHET10 | Medium brown | Present | 7.7 | 115.3 | 7.3 | Multinucleate | Brown | Immersed | 26 | Rough | |
| JHET11 | Hyaline to light brown | Present | 7.8 | 135.9 | 6.9 | Multinucleate | Medium to dark brown | Superficial | 26 | Smooth | |
| JHET12 | Dark brown | Present | 8.2 | 96 | 6.1 | Multinucleate | - | Immersed | 22 | Rough | |
| JHET13 | Medium brown | Present | 8.3 | 102 | 6.3 | Multinucleate | Medium to dark brown | Immersed | 38 | Rough | |
| JHET14 | Hyaline to light brown | Present | 6.9 | 135.9 | 7.3 | Multinucleate | - | Not present | 0 | Not present | |
| JHET15 | Dark brown | Present | 8.3 | 117.8 | 5.3 | Multinucleate | - | Superficial | 23 | Rough | |
| JHET16 | Medium brown | Present | 7.7 | 117 | 7.1 | Multinucleate | White to light brown | Immersed | 37 | Rough | |
| JHET17 | Hyaline to light brown | Present | 7.8 | 110.3 | 5.4 | Multinucleate | Light brown to dark brown | Superficial | 26 | Rough | |
| JHET18 | Hyaline to light brown | Present | 7.1 | 111.9 | 6.1 | Multinucleate | Medium to dark brown | Immersed | 45 | Smooth | |
| JHET19 | Dark brown | Present | 7.8 | 73.2 | 5.8 | Multinucleate | - | Superficial | 23 | Rough | |
| ISBT1 | Hyaline to light brown | Present | 6.8 | 86.7 | 5.8 | Multinucleate | Dark brown | Superficial | 45 | Rough | |
| ISBT2 | Medium brown | Present | 7.7 | 103.3 | 6.3 | Multinucleate | Medium to dark brown | Superficial | 39 | Rough | |
| ISBT3 | Medium brown | Present | 8.2 | 99.3 | 6.8 | Multinucleate | Medium to dark brown | Superficial | 50 | Rough | |
| ISBT4 | Hyaline to light brown | Present | 7.2 | 113.2 | 5.9 | Multinucleate | Dark brown | Superficial | 31 | Smooth | |
| ISBT5 | Medium brown | Present | 7.3 | 145.2 | 7.5 | Multinucleate | Light brown to dark brown | Superficial | 42 | Rough | |
